# Practical applicability of the STAMCO and ChOLE classification in cholesteatoma care

**DOI:** 10.1007/s00405-020-06478-7

**Published:** 2020-12-18

**Authors:** Fleur A. ten Tije, Paul Merkus, Joeri Buwalda, Henk M. Blom, Sophia E. Kramer, Robert Jan Pauw, DCD Consortium, Heike J. Nyst, Lisa van der Putten, Annemarie P. Graveland, Gerben G. Kingma, Jeroen W. L. van Lange, Guido T. M. de Kuyper, Johan M. Schmidt, Jantine Venker, Dick J. Warmerdam, Tjasse D. Bruintjes, Raphael J. B. Hemler, Kees J. Langenhuijsen, Stephanie M. Winters, Jeroen Mud, Adriaan F. Holm, Ferdinand A. W. Peek, Jan Pieter Koopman, Steven J. H. Bom, Henri-Jacques Tjong-Ayong, Jan Pieter de Mönnink, Nynke Boelstra van Cruijsen, Jurjan R. de Boer, Sietske F. Meinesz, Josephina M. Kruyt, David R. Colnot, Jasper J. Quak, Pepijn A. Borggreven, Rick van de Langenberg, Adriana J. G. E. Wedler-Peeters, Jorien Snel-Bongers, Eelco E. Kummer, Annette J. ter Schiphorst

**Affiliations:** 1 Otolaryngology-Head and Neck Surgery, Ear and Hearing, Amsterdam UMC, Vrije Universiteit Amsterdam, Amsterdam Public Health Research Institute, De Boelelaan 1117, Amsterdam, Netherlands; 2 Department of Otolaryngology-Head and Neck Surgery, Deventer Ziekenhuis, Deventer, The Netherlands; 3 Department of Otolaryngology-Head and Neck Surgery, Haga Ziekenhuis, Den Haag, The Netherlands; 4 Department of Otolaryngology-Head and Neck Surgery, Erasmus University Medical Center, Rotterdam, The Netherlands

**Keywords:** Cholesteatoma, Pathology, Classification, STAMCO, ChOLE

## Abstract

**Background:**

To compare cholesteatoma care internationally and to evaluate outcomes, ear surgeons must use the same terminology. However, a clear universal definition on how to describe the extension, destruction and accompanying morbidity caused by the cholesteatoma is lacking. The practical applicability by means of interrater agreement is assessed for the STAMCO and the ChOLE classification.

**Methods:**

A total of 134 adult patients derived from the nationwide multicentre study in the Netherlands, entitled Dutch Cholesteatoma Data (DCD) were included. Retrospective analysis of 134 surgical reports according to the STAMCO and ChOLE classification for localisation/extension of the cholesteatoma, complication status and ossicular chain status. Both the percentage agreement and the interrater agreement were determined for each item of the classifications and interrater agreement was compared between the classifications as a whole.

**Results:**

Differences in interrater agreement were found for both the localisation/extension of the cholesteatoma and ossicular chain status. STAMCO classification derived from the surgical report scored better on the localisation/extension of the cholesteatoma, whereas the ChOLE classification derived from the surgical report scored better on the status of the ossicular chain. In both classifications, complication status had a low agreement level but was also poorly registered in the surgical reports.

**Conclusion:**

Both STAMCO and ChOLE will be beneficial in uniform registration of cholesteatoma pathology in practice. Modifications proposed for both classifications may make them even more practical applicable in the future. A common denominator obtained from these two classifications may be incorporated in a standardised surgical report to facilitate evaluation which make outcomes transferable towards both classifications.

**Electronic supplementary material:**

The online version of this article (10.1007/s00405-020-06478-7) contains supplementary material, which is available to authorized users.

## Introduction

The ultimate goal for the ear surgeon in cholesteatoma care is to eradicate the disease, to prevent recurrent disease (a ‘safe’ ear), to create a functional self-cleaning, dry and waterproof ear, and to remain or restore hearing resulting in an acceptable generic and Chronic Otitis Media (COM) with cholesteatoma disease-specific quality of life. Analysing and comparing outcomes may help to improve results, predict outcomes, facilitate monitoring and feedback to otologists and to facilitate the discussion on treatment options during preoperative counselling. To facilitate the analysis, the comparison of results and to evaluate outcomes, otologists must use the same terminology to describe cholesteatoma pathology, surgery, outcomes measures and follow-up. However, due to worldwide differences, clear agreement on how to describe the extension, the destruction and accompanying morbidity caused by the cholesteatoma is scarce [1]. This makes it difficult comparing cholesteatoma outcomes in the literature.

In 2010, the Japanese Otological Society (JOS) published a paper on cholesteatoma classification and staging, in which they described how the middle ear and mastoid can be divided by means of the PTAM system and how to stage the cholesteatoma based on the extension in the tympanomastoid space [2].

Recently, the European Academy of Otology and Neurotology (EAONO) collaborated with the JOS and published a cholesteatoma classification based on an international consensus, in which the cholesteatoma is classified and staged by means of the STAM classification [3]. A Dutch cholesteatoma study, entitled the Dutch Cholesteatoma Data study (DCD), modified and implemented the STAM classification to STAMCO, separating complication status from the cholesteatoma location(s) and adding the ossicular chain status [4]. Almost simultaneously, the Swiss Society of Otolaryngology-Head and Neck Surgery introduced their classification ChOLE [5].

The STAMCO and the ChOLE classification both have the same items in their classification structure namely: localisation/extension, complication status and ossicular chain status. However, there are some differences between these two classifications. In the location/extension component of the STAMCO classification, the cholesteatoma can originate or (residual disease can) be located in either of the five divisions of the middle ear or mastoid. In contrast, in the ChOLE classification, all cholesteatomas originate in the tympanic cavity from where the extension continues. Drawings illustrate the separate locations in STAMCO and the extension and scoring system in ChOLE. The time point of assessment of the ossicular chain status differs. The ChOLE classification assesses the ossicular chain status after surgical removal of the cholesteatoma (but also after possible removal of affected or unaffected ossicular chain parts) all before the start of ossicular chain reconstruction. In STAMCO, the affected number of ossicles is described, reflecting the impact of the pathology, making registration of the exact pathology (localisation/extension) the most important and comparable factor. The decision of the surgeon to remove a certain ossicle is within the O of ChOLE and not within the O of STAMCO. Each denominator for ossicular chain status, will have a different association with hearing outcome as a whole and therefore are not exactly comparable. Drawings illustrate the ossicular chain status in ChOLE. Lastly, the ChOLE classification has pneumatisation and ventilation of the mastoid as one of the items of the classification, STAMCO does not incorporate this item.

Until now, none of the present classifications is widely adopted. One of the factors that determines the success of a classification is the practical applicability, because a higher practical applicability enhances the chance of implementation in daily practice. To achieve practical applicability, a high rate of interrater agreement between users of the same classification is demanded. This interrater agreement per classification can be determined using a concordance measure such as Fleiss’ Kappa [6].

Therefore, the aim of this article was to determine the practical applicability of the STAMCO and ChOLE classification by assessing the interrater agreement.

## Methods

For this study, four ENT surgeons (JB, HB, PM, RJP) with 8–21 years of experience in cholesteatoma surgery retrospectively scored surgical reports. From now on these four ENT surgeons are called raters. The raters score surgical reports which were retrieved from sixteen hospital based Otolaryngology centres spread across the Netherlands (2 university and 14 regional medical centres) within the national multicentre study conducted in the Netherlands, entitled the Dutch Cholesteatoma Data study (DCD). After retrieval, the surgical reports were stored in the database created in Castor EDC^™^ (Amsterdam) next to demographic, surgical, control examination and follow-up data. The raters retrospectively scored a total of 134 surgical reports for both the STAMCO and the ChOLE classification. They were simultaneously instructed before scoring in the use of the STAMCO and ChOLE classification and had no access to other information from the medical records of the same patient(s). Raters were aware of the fact that their scorings would be compared and during scoring no communication between raters was allowed.

All surgical reports obtained were from cholesteatoma patients with the following inclusion criteria: (a) > 18 years of age; (b) Patients who underwent surgery for primary, recurrent or residual cholesteatoma within the last year. Written informed consent was obtained of all patients prior to study participation. Other criteria for the complete study but not important for the presented data (c) good Dutch language proficiency of patients; (d) not pregnant and able to undergo a MRI. The study was approved by the Medical Ethical Committee of the VU University Medical Centre, Amsterdam; the Netherlands (reference number 2016/997) and local approval of the board of directors was received from the 16 participating hospitals.

After inclusion, patients were de-identified by assigning a number code to each patient.

### STAMCO

**Fig. 1:**
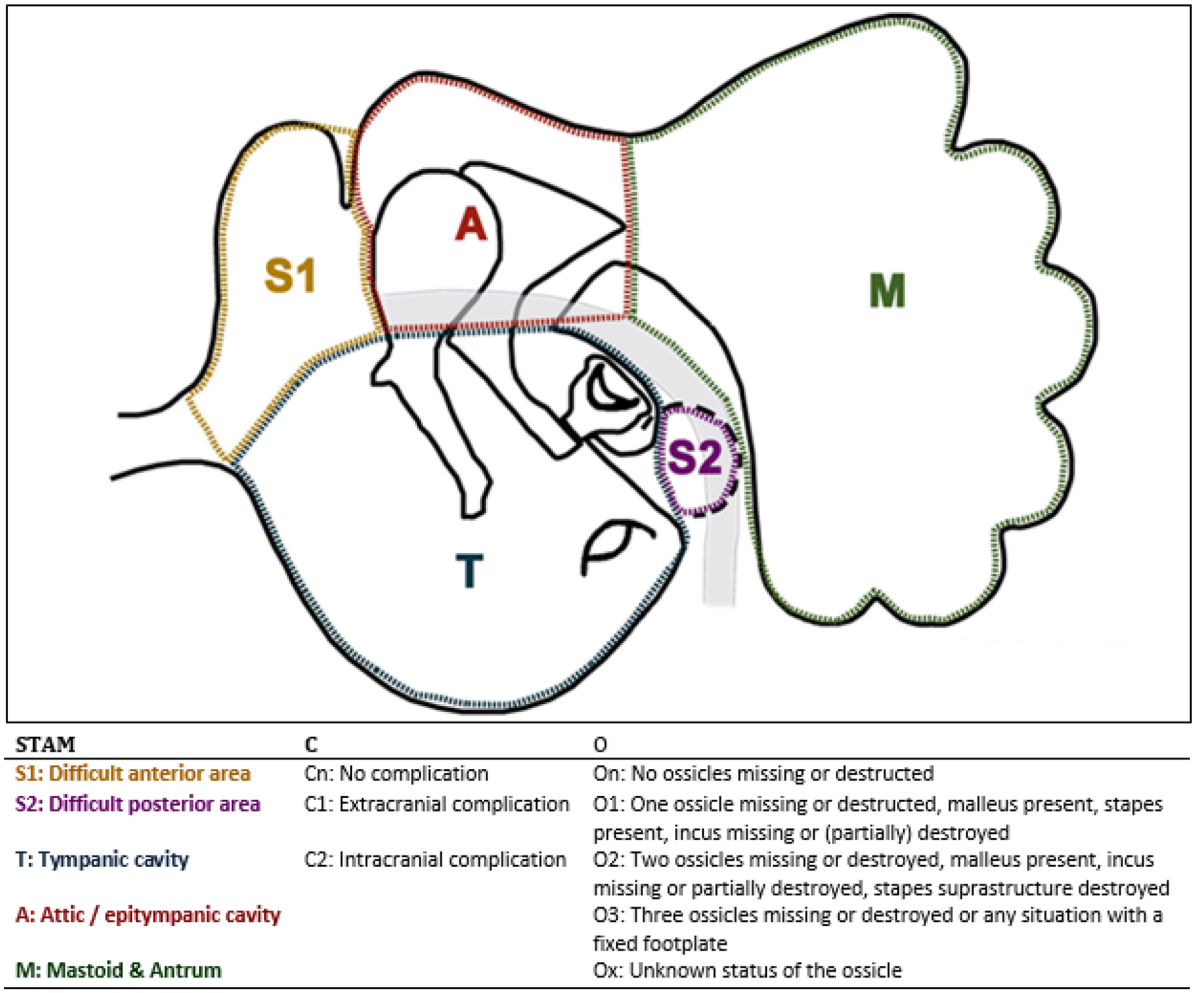
STAMCO Classification. The middle ear/mastoid is divided into five divisions (STAM). Complication status caused by cholesteatoma (**c**) can be divided into three categories and ossicular chain status (O) into five categories. Used with permission of the author and journal of international advanced otology. Merkus P, Tije FA, Stam M, Tan FML, Pauw RJ. Implementation of the “EAONO/JOS Definitions and Classification of Middle Ear Cholesteatoma” from STAM to STAMCO. J Int Adv Otol 2017; https://doi.org/10.5152/iao.2017.4049

STAMCO was scored by means of a scoring form and the raters also had access to Fig. 1. The STAMCO items were: 1. Description of the localisation/extension of the cholesteatoma 2. The presence of preoperative complication status and 3. The ossicular chain status before/ at the time of the removal of the cholesteatoma. To allocate the localisation/extension of the cholesteatoma, the middle ear and mastoid was divided into five divisions, which are: difficult access site 1 (S1, the supratubal recess), difficult access site 2 (S2, the sinus tympani), tympanic cavity (T), attic (A), and mastoid and antrum (M). These five divisions were mentioned separately on the scoring form and the raters had to click on either the yes or the no box for presence of cholesteatoma in one or more divisions. Complication status could either be intracranial (for example: meningitis)—or extracranial (for example: facial palsy) or no complications (prior to surgery) present. The raters had to click either the no complication or intra- or extracranial box on the scoring form. The ossicular chain status was assessed in five categories (See Fig. 1). The box of the observed ossicular chain status had to be clicked on the scoring form.

### ChOLE

From the surgical report the ChOLE items were extracted and scored according to the manual on the ChOLE website (https://chole.surgery accessed: 14-07-2019). These items were: localisation/extension (Ch), ossicular chain status at the end of surgery (O) and complication status (L). The degree of pneumatisation and ventilation (E) was not assessed.

### Percentage and Interrater agreement STAMCO and ChOLE

First, degree of agreement in percentages was calculated for each of the three items in both classifications separately: localisation/extension of the cholesteatoma, complication and the ossicular chain status. Localisation versus extension: in ChOLE, the word “extension” is used. As they classify according to the growth patterns of cholesteatoma and always have the middle ear as their starting point. This makes this classification useful for describing primary cholesteatoma.

In the STAMCO classification the area that is affected with cholesteatoma is called “localisation”. This is because also a recurrent or residual cholesteatoma can be classified with this system and usually in cases with a residual cholesteatoma, the localisation is described instead of the extension. In this paper, we use localisation/extension to indicate the same domain that is investigated in both classifications. Agreement on an item was defined as three or four raters (75 or 100%) scoring the same on that item of the surgical report. The degree of agreement per item in percentages was calculated by dividing the number of surgical reports with agreement by the total number of assessed surgical reports. For example, if in 90 reports out of the 134 surgical reports the majority of the raters agree a percentage of 67% agreement (90/134 × 100) is calculated for that item. Because the item localisation/extension of the STAMCO classification is divided into five categories, the mean was taken from these five categories to obtain an overall score.

Next to this, Fleiss’ Kappa tests were performed for each item separately for both the STAMCO and ChOLE classification to assess the interrater agreement, taking into account the agreement that is expected to occur by chance. The Fleiss’ Kappa and their 95% confident intervals were calculated using SPSS statistical package version 24 (SPSS Inc, Chicago, IL) based on multiple raters (*n *= 4) and nominal data [7]. The greater the Kappa value, the higher the interrater agreement [8]. According to the literature, the strength of agreement for the Fleiss’ Kappa was interpreted as poor (Kappa values < 0); small (0.0–0.20); fair (0.21–0.40); moderate (0.41–0.60), substantial (0.61–0.80) and almost perfect (0.81–1.00) [9].

## Results

**Fig. 2:**
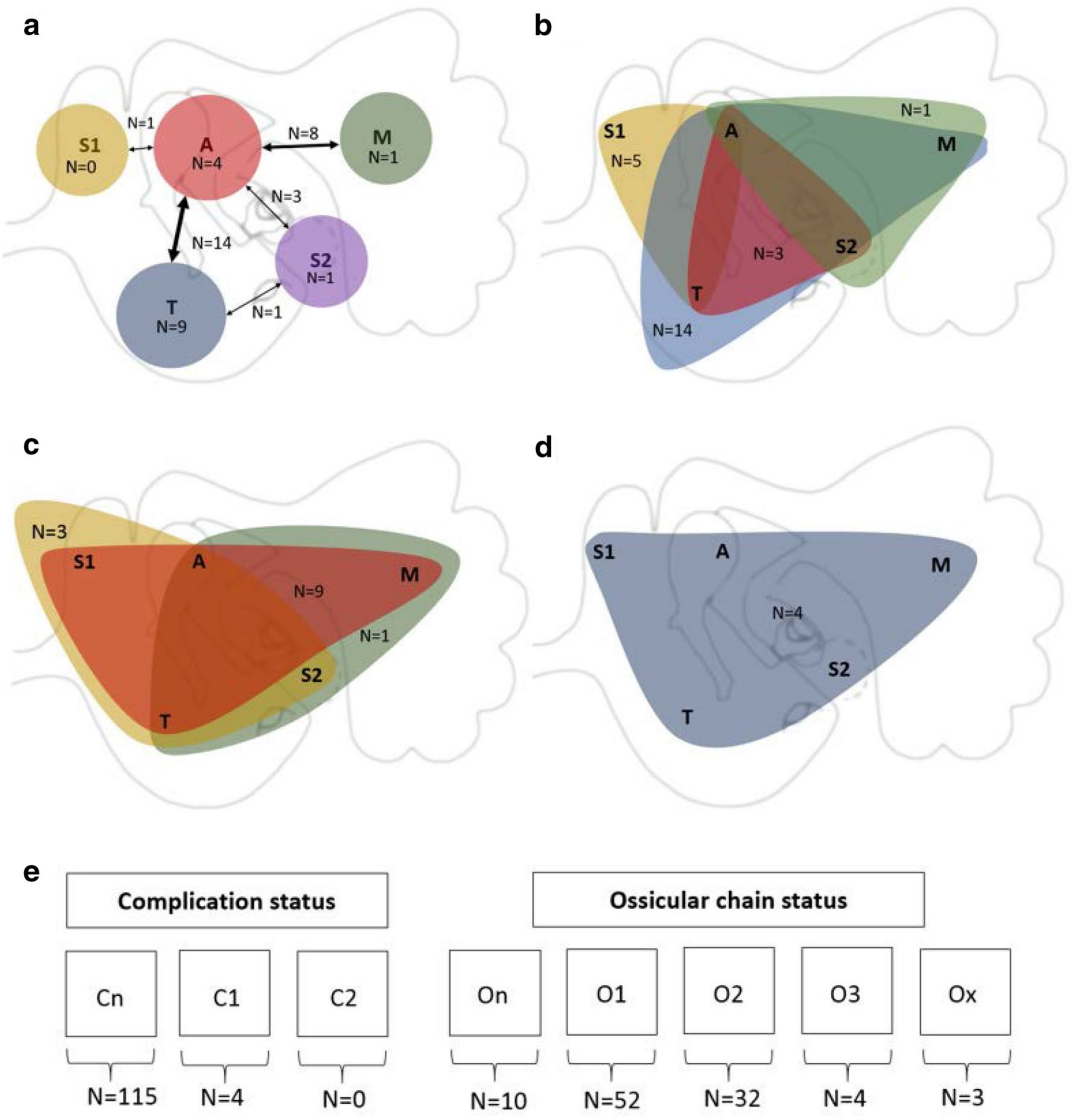
**a–e** Cohort pathology characteristics STAMCO. **a** shows the number of patients with a (smaller) cholesteatoma present in one location (circles) and two locations (arrows). **b** shows the number of patients with cholesteatoma present in three locations. **c** shows the number of cholesteatoma present in four locations. **d** shows the number of patients with cholesteatoma present in five locations. Figure **e** shows the number of each complication status and each ossicular chain status at the start of the surgery

From the 134 surgical reports, 7 could not be analysed due to an incompleteness of the surgical report and were excluded. A total of 127 surgical reports were analysed by the 4 raters. In Fig. 2, the cohort pathology characteristics of the STAMCO classification are shown. If there was no agreement (agreement is when three or four raters have a similar scoring) on one or more of the categories of the localisation/extension item, this surgical report was excluded for this figure. In total 109 surgical reports had an agreement of 3 or 4 raters for localisation (85%), 119 for complication status (94%) and 93 for the status of the ossicular chain (73%). The ChOLE classification had an agreement of 3 or 4 raters in 96 surgical reports for localisation (76%), 113 for complication status (89%) and 113 for the status of the ossicular chain (94%).

In most cases, no agreement on the localisation/extension item in the STAMCO classification was observed on involvement of location T (tympanic cavity) when cholesteatoma was present in location A (attic/epitympanic cavity). The lack of agreement observed on the complication status were predominantly seen when the raters had to choose between no complication and extracranial complication. E.g., two of the raters scored an open semi-circular canal as no complication instead of an extracranial complication.

No agreement was often observed on the ossicular status between option O2 and O1. In the ChOLE classification, most cases with no agreement on the localisation/extension item was observed on involvement of the sinus tympani and/or the supratubal recess. No agreement was often observed on the complication status between no complications and extracranial complication. And no agreement on the ossicular chain status was often observed between option O1 and O2 and between option O2 and O3b.

In Fig. 2 the characteristics of the cohort according to the STAMCO classification is graphically presented. In 15 patients, cholesteatoma was present in 1 location (Fig. 2a). No cholesteatoma was found solely in location S1. In total, 27 combinations of cholesteatomas present in 2 locations were identified in this cohort (Fig. 2b). In addition, 23 combinations of cholesteatoma present in 3 locations and 13 combinations of cholesteatoma present in 4 locations were identified. In four patients, cholesteatoma was present in all locations of the middle ear and mastoid. In 115 patients, no complication status was reported by the raters. One ossicle missing or destroyed (O1) was the most observed status of the ossicular chain, followed by two ossicles missing or destroyed (O2) followed by ossicle chain intact (On).

The level of agreement (in percentages) between the four raters, were calculated for both the STAMCO and the ChOLE classification, are shown in Table 1.

**Table 1:** Level of agreement in percentages of the three items

Items	STAMCO	ChOLE
Localization/extension	85%	76%
Complication status	94%	89%
Ossicular chain status	73%	94%

The Fleiss kappa scores with 95% confidence intervals were calculated for both STAMCO and the ChOLE classification, which are shown in Table 2.

**Table 2:** Fleiss kappa scores and degree of agreement on each item of both the STAMCO and ChOLE classification

	Fleiss Kappa Score	95% confidence interval	*p* value
**STAMCO**			
Localization/extension	0.58 (moderate)	0.50–0.66	(*p* < 0.0001)
Complication status	0.27 (fair)	0.19–0.34	(*p* < 0.0001)
Ossicular chain status	0.47 (moderate)	0.42–0.52	(*p* < 0.0001)
**ChOLE**			
Localization/extension	0.26 (fair)	0.22–0.30	(*p* < 0.0001)
Complication status	− 0.09 (poor)	− 0.15– − 0.03	(*p* < 0.0001)
Ossicular chain status	0.65 (substantial)	0.60–0.70	(*p* < 0.0001)

The strength of agreement for the Fleiss Kappa was interpreted as poor (Kappa values < 0); small (0.0–0.20); fair (0.21–0.40); moderate (0.41–0.60), substantial (0.61–0.80) and almost perfect (0.81–1.00)

On localisation/extension of the cholesteatoma, the STAMCO classification has a “moderate” kappa score (0.58). This is a higher kappa score in comparison to the “fair” kappa score (0.26) of the localisation/extension of the ChOLE classification. On complication status, both classifications do not score a high level of user agreement. STAMCO scores a “fair” kappa score (0.27) and ChOLE has poor agreement (< 0).

On the ossicular chain status, the STAMCO classification has a “substantial” kappa score (0.65) compared to a “moderate” score (0.47) of the ChOLE classification.

The P values shown in Table 2 indicate that the expected user agreement between raters is significantly better than would be expected by chance, thus the degree of association is better than expected. No *t *tests have been performed due to differences in the categories of all items in both classifications.

## Discussion

The study was carried out to investigate how well the cholesteatoma classification systems STAMCO and ChOLE are applicable to register cholesteatoma pathology in practice using a Dutch multicentre cohort. This practical applicability is measured by means of the interrater agreement for both the STAMCO and the ChOLE classification. Although classifications like these are designed for prospective use, the current study may help to understand the difficulty between current practice and implementation of a new classification. It is in the interest of all ENT surgeons to evaluate and work towards a widely used classification system. Adaptations and improvements should be considered and tested even once a classification has been published. The results of this study should be seen in this light.

From the results, we can conclude that STAMCO and ChOLE are applicable to retrospectively register cholesteatoma pathology in practice. We will discuss each found item separately. For localisation/ extension STAMCO has a higher interrater agreement score compared to ChOLE. A possible explanation is that in the ChOLE classification all cholesteatomas originate from the tympanic cavity. This hinders use of the ChOLE classification in the follow-up of cholesteatoma patients with a residual cholesteatoma, since residual cholesteatoma can be found in any localisation separately. The STAMCO classification is applicable for primary, recurrent and residual cholesteatoma and is thus suitable for registration of cholesteatoma during follow-up.

A second possible explanation why ChOLE scores less on localisation is the grey area between Ch score 1 (Middle ear) which is not very clear divided from a score 2 (Middle ear and Attic).

STAMCO has a higher interrater agreement for complication status compared to the ChOLE classification, but has a lower score for ossicular chain status. These differences were not assessed statistically due to differences in the categories of the three items of both classifications. The lower score for the ossicular chain status of the STAMCO system could be due to the used ambivalent terms, which is either missing or destructed. The ChOLE system only has one term (‘missing’). On the other hand, to classify also the removed ossicle(s), in the ChOLE classification, the judgement or preference of the surgeon is included in the classification. Furthermore, both the STAMCO and ChOLE classification had a low kappa score for the complication status (the complication due to the cholesteatoma not because of the surgery). A possible explanation for this is that the STAMCO classification does not contain a Cx (not identifiable) in the complication status item. It might be possible that if STAMCO did contain Cx, both kappa values would have been equal. If the complication status is not mentioned in the surgical report, raters are not able to answer the question. However, reporting a complication status can indicate the impact/severity of the cholesteatoma and with this also the difficulty of the surgery or the decisions during surgery and is therefore important information to report in the surgical report. In addition, retrospectively scoring complication status is difficult due to a low incidence of complications caused by the cholesteatoma before surgery. Two of the raters scored a dehiscent semi-circular canal as no complication instead of an extracranial complication. This could have been due to misinterpretation of the terminology. The problem occurred with the word dehiscent, which could be interpreted as an open canal and therefore a complication or partly covered and therefore no complication. This is something which could be improved in standard classifications, through clearly defined definitions and uniformly used terminology. The degree of pneumatisation and ventilation (E), like in ChOLE, was not be assessed, because this was not mentioned in the surgical reports and is not an item of the STAMCO classification. Also infected versus non-infected; infiltrative versus non-infiltrative and a well-pneumatised vs poorly pneumatised mastoid are items of discussion to be included in a classification. For research purposes, it seems beneficial to include these factors in the surgical files or databases, but to get a start with an accepted classification in all centres this may be too much ‘registration burden’. The main outcomes of cholesteatoma surgery are in short defined as: presence/absence of cholesteatoma; hearing outcomes and complaints of patients [10].

The most prominent limitation of the study is the retrospective scoring of surgical reports instead of prospective scoring, or scoring of operation videos, since scoring in a prospective manner is the intended method for these systems. However, it seems the best possible method to assess different classification systems at this moment because of the practical limitations of using two scoring systems simultaneously. Another drawback was the variable quality and completeness of the surgical reports, which was the result of using the multicentre cohort with many different surgeons. Additional studies to assess both classification systems prospectively are warranted, but must take note that the surgeons are properly instructed and are familiar with both classifications. This will result in surgical reports that contain the required elements for both STAMCO and ChOLE classification systems, thereby overcoming the above mentioned limitations of the present study. The raters in our study were equally experienced in the use of the STAMCO and ChOLE classification. Therefore, we assume that there was no bias towards the correct use of both classifications.

Next to registration of the standard elements in the surgical report, it is of importance to register pathology, surgery, outcome measures and follow-up as mentioned in the introduction. The pathology could be registered by means of the above mentioned classifications. The surgery could be registered and could contain elements from the consensus-based categorization of tympanomastoid surgery SAMEO-ATO [11]^.^ Outcome measures like the presence/absence of residual or recurrent cholesteatoma, hygienic status and hearing must be registered, but also the method used for the follow-up. However, to have a complete picture, patient reported outcome measures (PROMs) should also be registered [12] . Different Health-Related Quality of Life (HR-QOL), hearing and otological questionnaires are available [13–17]. The main reason for using, the domain-specific OQUA in this study is because of its ability to assess the impact of each complaint separately. Questions about earache, pressure sensation, itching, tinnitus, hearing loss, ear discharge, loss of taste and dizziness complaints can all be investigated separately during follow-up. It should be mentioned that the more disease-specific questionnaires that could have been used in this study are the two tools designed by Philips, which are the COMQ-12 and COMBI for chronical otitis media with or without cholesteatoma [16, 17]. A Dutch translation of both questionnaires is available and validated [18–20]. Because of the overlap in questions, some language issues, and the many questionnaires already in our study we have decided not to use these questionnaires. Still, for future research and to assess patient-reported outcomes, the COMQ-12 and COMBI are probably the best option in cholesteatoma care as they are widely used and have a high number of validated translations in different languages. To register hearing, the Amsterdam Hearing Evaluation Plots (AHEPs) can be very beneficial. In this plot, both bone- and air conduction pre- and postoperatively are presented to evaluate change in hearing after surgery. In addition, the safety of the procedure regarding preserving inner ear function can be assessed [21]. Next to HR-QOL, hearing and otological questionnaires, vestibular function should be questioned and with complaints further tested.

Another limitation is within Fig. 2, where not all 127 surgical reports are displayed. A fair amount of surgical reports were excluded from the dataset to form this figure. The reason for excluding surgical reports to build this figure was because only the items with agreement of at least three raters were included. Therefore, combinations with agreement of the localisation/extension of the cholesteatoma are displayed in Fig. 2.

As displayed in the cohort pathology characteristics STAMCO overview, in eight cases there was no agreement on complication status. This could be due to the terminology that is used for complication status. The term ‘complication status’ could be interpreted as a complication during surgery instead of a complication preceding the surgery (a “complicated case” or a “complication caused by the cholesteatoma” like a dehiscence of the lateral canal or a facial nerve palsy prior surgery). A modification of the term is necessary to obtain a clear definition in both classification systems. We propose to alter complication status into complication status caused by the cholesteatoma, indicating complications that arise in the period preceding the surgery.

The status of the ossicular chain is an important factor in the analysis of the hearing outcome, therefore this must be registered in a classification system. As mentioned in the paper of Merkus et al. reporting the ossicular chain status is important because it reflects the impact of the cholesteatoma and some ossicles are more important in hearing reconstruction than others. In addition, scoring this item makes it possible to correlate the extent of the disease with the hearing related outcomes. Due to these arguments, the alphabetical character O was added to the STAM classification to form STAMCO [4]. As previously mentioned, the time point of registration of the ossicular chain status differs between the two classifications and may reflect two different aspects, namely the destruction of the cholesteatoma (STAMCO) and the ossicular situation created before reconstruction (ChOLE). But there is more than a difference from a surgical time point. STAMCO is intended to compare cases with similar destruction of the ossicular chain, thus based on the extent of the pathology, whereas the ossicular chain status of ChOLE is a result of both the extent of the pathology as well as choices of the surgeon. There is a difference in interrater agreement concerning the status of the ossicles between the STAMCO and the ChOLE classification, but it is unknown if this leads to a good advice which ossicle status registration is most useful. The moderate agreement for this item of the STAMCO classification could be an argument for not registering the ossicular chain status when using this classification. However, the influence of the ossicular chain status at any of the two time points on the outcome of hearing is still unclear. Adjusting the ossicular chain status item could enhance interrater agreement and with it, the practical applicability. First of all, a clear definition with regard to the different categories within the ossicular chain status should be formed. Currently, the definition of all the five categories are “missing or destructed” is too vague. A partially eroded ossicle is weighed in the same extent as a totally destructed ossicle namely O1. But a destructed incus or stapes, for example, probably has a bigger impact on hearing outcome than a partially eroded malleus head. Which ossicle is destructed and if this ossicle remainder is still useful without removal, are essential elements. An example would be the eroded long process of the incus, in which the complete incus could be removed or the remainder preserved and used in an incus stapes connection (with cement or prosthesis). Both situations could influence either residual rate or hearing outcomes.

Therefore, we propose to register the ossicular chain status at these two different time points (initial destruction and removal), until we can correlate these data with hearing outcomes or the number of residual or recurrent cholesteatoma. The drawings of the destruction or missing ossicles of the ChOLE could be useful in this respect, as long as it represents the situation before removal of ossicles. The O1 drawing represent two situations (long process erosion and complete incus destruction/removal), which could lead to two or more therapeutic options with different hearing outcomes [22, 23]. To cover both situations both drawings will be useful as 1a (incus long process erosion), 1b (majority of incus destructed). The drawing of ChOLE O2 could be argued the same, although we are not aware of any different surgical option between both situations. The drawings of ChOLE may be added to the STAMCO classification to make the ossicular status more clear.

The Fleiss kappa method chosen in this study to measure practical applicability, by means of the interrater agreement is suited in this situation, because the surgical reports were analysed by more than three raters and are chosen randomly from a large population [24]. Next to this, the level of measurement is nominal. A pitfall of the used technique is that in some cases it may return low values (poor, small, fair) even though the percentage agreement is high [25]. For example in this study, the percentage agreement of the complication status is high for both classifications, but the interrater agreement is low (poor and fair). This is due to the fact that complication status is scored with a very high agreement but the Fleiss kappa test does not allow to discriminate between a perfect agreement and other situations. This paradox (high percentage agreement, low kappa score) can be avoided using Bootstrap confidence intervals, because this allows kappa to recognize a higher inter-rater agreement. This might be an option for future studies with a larger cohort. In addition, raters were aware of their scoring being compared. It could be that a Hawthorne effect may have altered rater’s behaviour and may have had a positive effect on the outcomes [26].

One of the purposes of a classification is to predict outcomes. This can be achieved by prospectively following up for a long period of time, whilst registering outcome measures [27]. Correlation of these outcomes with the classification, allows prediction of results and facilitates the development of a staging system. Both the STAMCO as the ChOLE have the possibility of staging. The STAM classification by the EAONO/JOS is setup as a staging system. In the consensus statement, it is mentioned that the STAM staging system reflects the severity of the disease, difficulty of complete removal and restoration of a normal function of the ear. But it could be that some items of the classification have a larger impact on staging then other items have. Because all information is converted into one single staging score, useful information is lost and hinders improvement of care. In the study of James et al., the validity of the EANO/JOS staging system was evaluated by means of an international collaboration. They state that the validity of the staging system is determined by how complete the necessary information needed was recorded. And a lack of clear correlation between the stage and risk of recurrent cholesteatoma is still a limitation [28]. Thus, before staging, it is important to gain more experience in using the classification and a longer period of registration. Therefore, we propose to postpone staging at this stadium in both classification systems. Next to registration, it is important to register the long-term outcome measures at least 5 years after surgery [29]. With these results, a survival analysis (disease free interval) by means of Kaplan–Meier tests can be performed to examine the course and recurrent character of cholesteatoma [30]. The hearing, both bone- and air conduction pre- and postoperatively must be registered to evaluate “safety” concerning the inner ear hearing status. The Amsterdam Hearing Evaluation Plots (AHEPs) could contribute in this type of hearing evaluation [18]. Throughout the whole follow-up period of at least 5 years checks for recurrence or residual must be performed by means of MRI and otoscopic consults [31].

Both classification systems are applicable to register cholesteatoma pathology in daily practice. After proposed modifications, like an altered location/extension of the ChOLE classification to also include residual cholesteatoma in the scoring system; an improvement of the ossicular chain status of the STAMCO classification by means of adding drawings of the different possible scenarios; a better description of pre-operative complication status in both classifications with examples, may enhance practical applicability even more. Next to this, further remodelling of both classification systems towards one universal accepted system could be done using video recordings, pre-operative high-quality Cone Beam CT-scans and MRI including non-EP DW MRI could be performed. If the separate items of both classifications are registered in the surgical report, a common denominator can be determined in the future. For an international database of cholesteatoma care in the future, information about the surgery and patient related outcomes must be registered next to studied classifications to register the pathology of cholesteatoma. Good examples with other disorders can be seen in work done by the International Consortium for Health Outcomes Measurement (ICHOM), in which a standard set of value-based patient-centered outcomes for this disorder are formed [32]. Recently, the Dutch ENT society has developed, with a Delphi consensus project, a set of outcome measures and context information for the registration of cholesteatoma care to create uniform data registration in the Netherlands. These outcome measures are (1) the presence/absence of a cholesteatoma in the first 5 years after surgical removal of cholesteatoma, (2) hearing level after surgical removal of cholesteatoma, and (3) the documented assessment of patient’s complaints with a validated patient reported outcome measures questionnaire (PROM). Furthermore, consensus was reached on the registration of cholesteatoma type (residual/recurrent), localisation and how to report the presence of cholesteatoma in the follow-up [10].

## Conclusion

A practical applicable classification system for uniform registration of the pathology is necessary to allow ENT surgeons to analyse outcomes, compare their results and facilitate monitoring and feedback. Both STAMCO and ChOLE will be beneficial in this matter, but can improve on certain aspects of the classification. A common denominator obtained from these two classifications may be incorporated in a standardised surgical report to facilitate evaluation and perhaps development of a staging system in the future.

## Electronic supplementary material

Below is the link to the electronic supplementary material.

Supplementary file1 (DOCX 110 KB)
