# Supplementary figures and images for: Practical applicability of the STAMCO and ChOLE classification in cholesteatoma care
Source: Eur Arch Otorhinolaryngol. 2020 Dec 18;278(10):3777–87. doi: 10.1007/s00405-020-06478-7 (PMC8382628; doi:10.1007/s00405-020-06478-7)

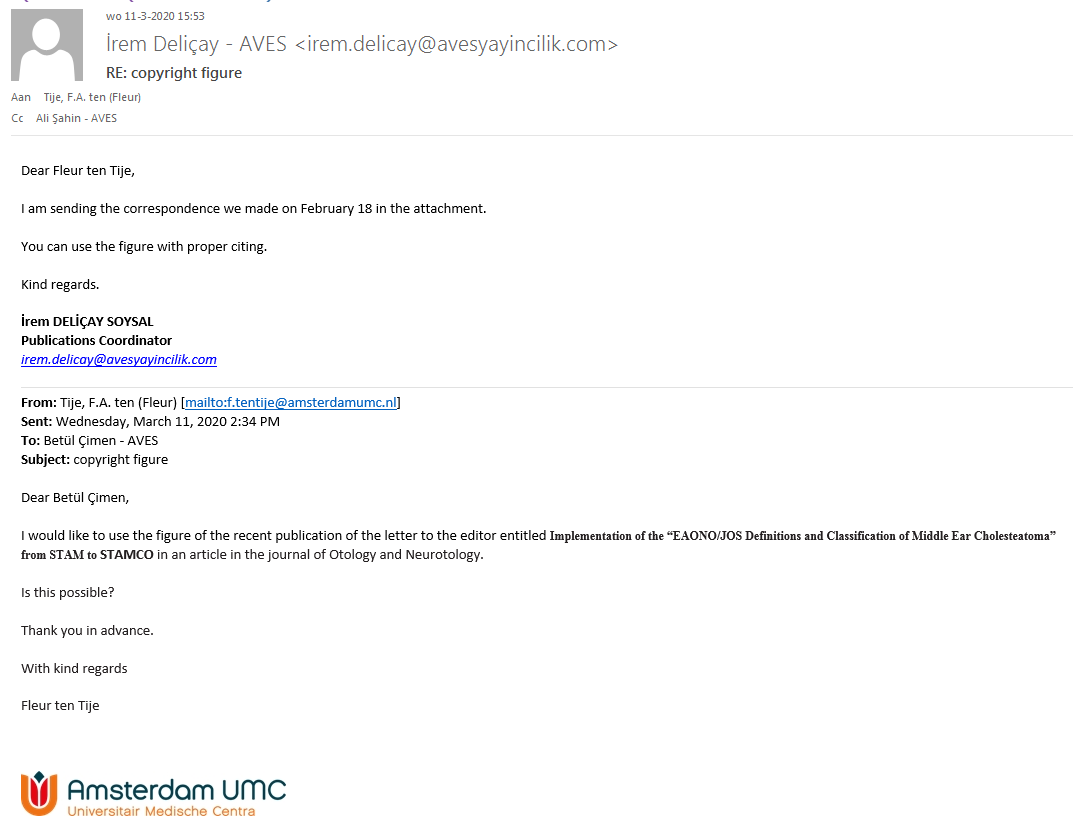


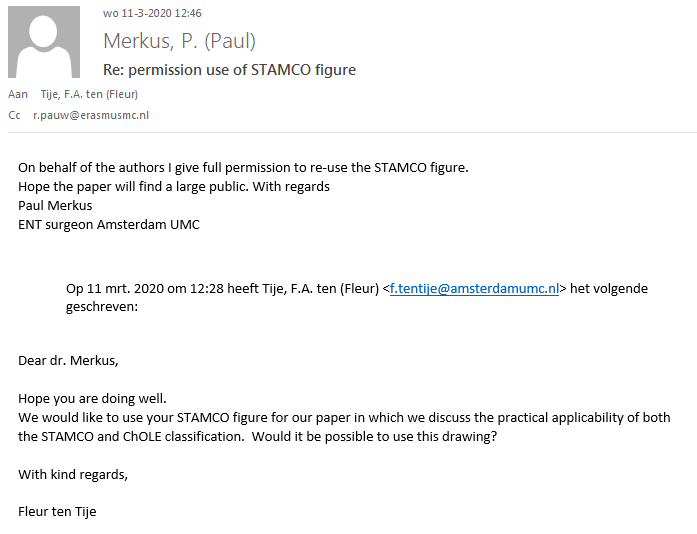

Supplement: Supplementary file 1 — Supplementary file1 (DOCX 110 KB) [file 405_2020_6478_MOESM1_ESM.docx]
